# Outcomes of balloon angioplasty and stent placement for iliac artery lesions classified as TASC II A, B: a single-center study

**DOI:** 10.3389/fsurg.2024.1366338

**Published:** 2024-03-27

**Authors:** Le Duc Tin, Lam Van Nut, Abdelrahman Sherif Abdalla, Hoang Duc, Patrick A. Kwaah, Trang T. B. Le, Tran Thi Thuy Vy, Thoa Le, Pham Minh Anh, Do Kim Que, Nguyen Tien Huy

**Affiliations:** ^1^ Department of Vascular Surgery, Cho Ray Hospital, Ho Chi Minh City, Vietnam; ^2^ Department of Thoracic and Vascular Surgery, Nam Can Tho University, Can Tho, Vietnam; ^3^ Department of Internal Medicine, AdventHealth, Sebring, FL, United States; ^4^ Department of Internal Medicine, Hanoi Medical University, Hanoi, Vietnam; ^5^ Cardiovascular Research, Methodist Hospital, Merrillville, IN, United States; ^6^ Department of Internal Medicine, Yale School of Medicine, Yale-Waterbury Internal Medicine Program, Waterbury, CT, United States; ^7^ Department of Internal Medicine, University of Medicine and Pharmacy at Ho Chi Minh City, Ho Chi Minh City, Vietnam; ^8^ Department of Internal Medicine, Minh Anh International Hospital, Ho Chi Minh City, Vietnam; ^9^ Department of Thoracic and Cardiovascular Surgery, Thong Nhat Hospital, Ho Chi Minh City, Vietnam; ^10^ Institute of Research and Development, Duy Tan University, Da Nang, Vietnam; ^11^ School of Medicine and Pharmacy, Duy Tan University, Da Nang, Vietnam; ^12^ School of Tropical Medicine and Global Health, Nagasaki University, Nagasaki, Japan

**Keywords:** iliac artery occlusion, balloon angioplasty, stent placement, TASC II A, TASC II B, endovascular interventions, short and mid-term outcome

## Abstract

**Background:**

Iliac artery stenosis or occlusion is a critical condition that can severely impact a patient's quality of life. The effectiveness of balloon angioplasty and intraluminal stenting for the treatment of iliac artery lesions classified as TASC II A and B was evaluated in this single-center prospective study.

**Methods:**

Conducted between October 2016 and September 2020 at Cho Ray Hospital's Vascular Surgery Department, this prospective study involved PAD patients categorized by TASC II A and B classifications who underwent endovascular intervention. Intervention outcomes were assessed peri-procedure and during short-term and mid-term follow-ups.

**Results:**

Of the total of 133 patients, 34.6% underwent balloon angioplasty, while 65.4% received stenting. The immediate technical success rate was 97.7%, while the clinical success rate was 62.4%. Complications were minimal, with major limb amputation reported in 1.5% of the cases. There was a significant improvement in Rutherford classification and ABI at short-term follow-up, with a patency rate of 90.2%. The mid-term post-intervention follow-up yielded similar results with an 86.1% patency rate. The mortality rates associated with arterial occlusion were 2.3% during short-term follow-up and 1.7% during mid-term follow-up.

**Conclusion:**

Balloon angioplasty and stent placement are effective and safe interventions for TASC II A and B iliac artery occlusions with favorable short and mid-term outcomes. Further, multi-center studies with larger sample sizes are recommended for more comprehensive conclusions, including long-term follow-up assessment.

## Introduction

Peripheral arterial disease (PAD) is a widespread concern, causing substantial health issues and mortality. Studies note its prevalence at 15%–30% in developed nations, with a significant burden, especially among the elderly (1, 2). Within the realm of PAD, aortoiliac occlusive disease poses a unique challenge, given their pivotal role in the lower limb circulation (3).

**Figure 1:**
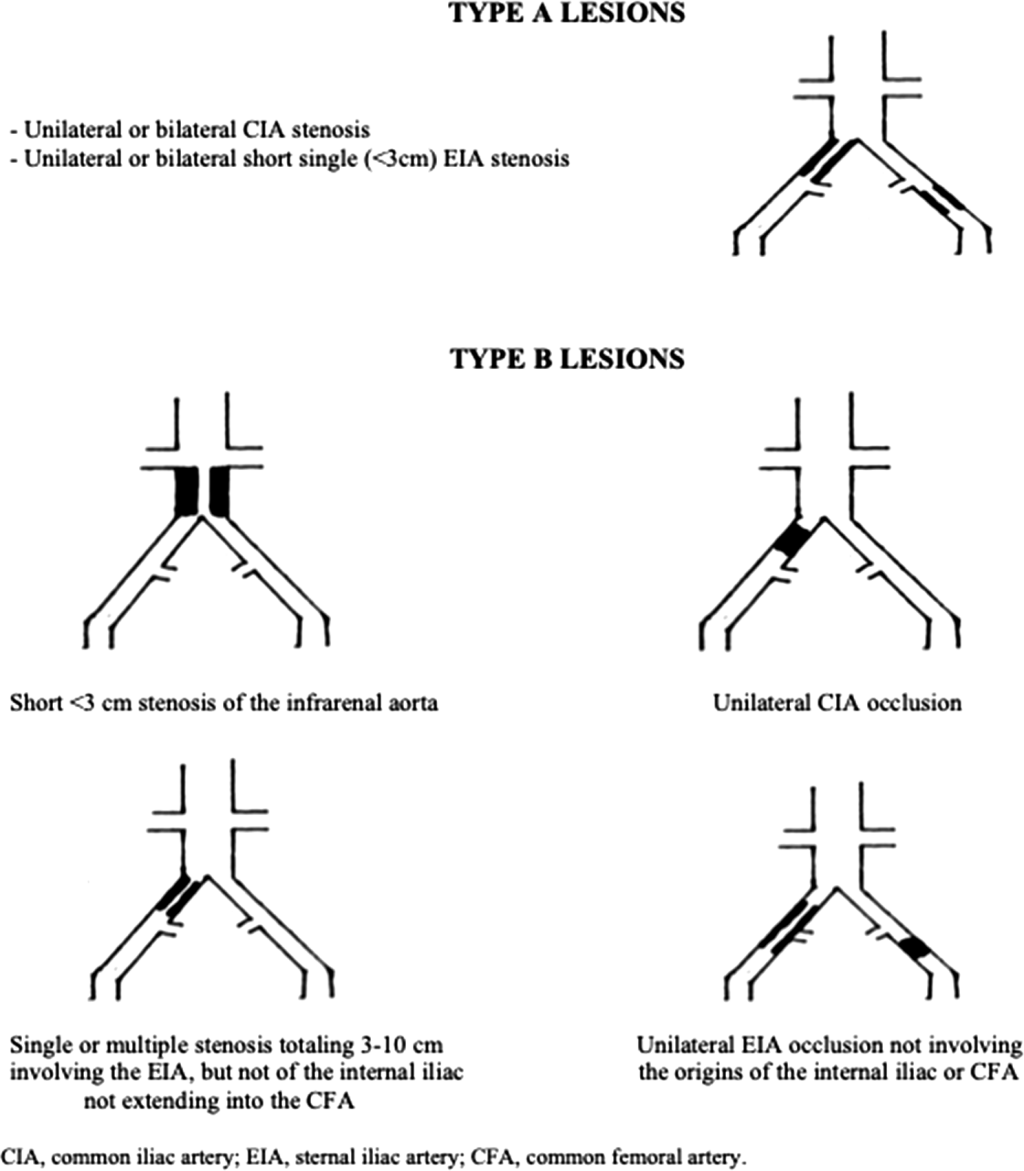
The TASC II A and B classification of aortoiliac lesions.

Iliac artery stenoses or occlusions are a common, critical aspect of peripheral arterial disease, significantly impacting life quality and posing management challenges. The TransAtlantic Inter-Society Consensus (TASC) classification system is a well-established framework utilized in the classification and treatment of PAD, particularly in the lower extremities. TASC II (the second iteration of the classification) further refines the categorization of iliac artery lesions based on their severity and complexity (4). TASC IIA involves limited iliac artery segments, potentially involving the common iliac artery or the external iliac artery. On the other hand, TASC IIB denotes a different category of iliac artery lesions, which are typically more extensive and may extend into the common femoral artery (Figure 1). These two categories are generally amenable to endovascular treatments, which entail minimally invasive catheter-based procedures. This classification has enhanced our understanding of the disease, yet many aspects, such as the optimal treatment modality for TASC II A, B occlusions, remain subject to ongoing debate (4).

Among the endovascular options, balloon angioplasty and stent placement have emerged as prevalent and promising treatment modalities. Balloon angioplasty, a technique wherein a balloon catheter is used to open up the occluded artery, has proven effective in many cases (4, 5). However, the development of stents, which provide structural support to the artery, has introduced an alternative option (5, 6). While both of these approaches have their merits, evaluating their comparative effectiveness and short-to-midterm outcomes is warranted.

In our study, we embarked on an assessment of the short and medium-term outcomes of balloon angioplasty and stent placement for treating iliac artery lesions categorized as TASC II A, B. Our objective is not only to provide a comprehensive comparison of their effectiveness, but also to identify potential determinants of these outcomes, shedding light on potential indicators of treatment success. Focusing on short-term and medium-term results enables a more comprehensive understanding of the longevity of these procedures, extending our comprehension beyond immediate post-operative outcomes. This research, therefore, seeks to bridge existing knowledge gaps, equipping clinicians with evidence-based insights to inform therapeutic choices and refine personalized treatment strategies, ultimately enhancing overall patient management efficiency.

## Methods

### Study design and participants

The reporting of this study followed the Strengthening the Reporting of Observational Studies in Epidemiology (STROBE) guidelines for reporting observational studies, and the checklist is available. The prospective study spanned from October 2016 to September 2020 and involved patients diagnosed with PAD at the Department of Vascular Surgery, Cho Ray Hospital. Following their selection, these patients underwent endovascular intervention, with subsequent follow-ups conducted at intervals of 1, 3, 6, 12, 24, and 36 months post-intervention. Inclusion criteria for the study were as follows: patients must exhibit symptoms of chronic lower limb ischemia as per the Rutherford classification, encompassing intermittent claudication impacting daily activities (Rutherford grades 2, 3), rest pain (Rutherford grade 4), and non-healing ulcers or gangrene (Rutherford grades 5, 6). In addition, participants needed to have isolated TASC II A, B iliac artery lesions, regardless of the presence of any associated femoral artery lesions.

Patients were excluded from the study if they had TASC II A or B iliac artery lesions accompanied by Rutherford grade 1 or above-the-knee amputation, or if these occlusions were associated with a high surgical risk, as defined by the American Society of Anesthesiologists (ASA) grades IV and V. Patients who had previously undergone hybrid surgical and endovascular interventions for TASC II A or B iliac artery occlusions, or those who did not provide consent to participate in the study, were also excluded.

### Sampling

Sampling formula for estimation of hazard ratio was used to calculate the sample size:

$$n\geq \;\frac{\sigma^{2}{\left(Z_{1-\;\alpha /2}+Z_{1-\beta }\right)}^{2}}{{\left(\log_{e}\mathrm{HR}\right)}^{2}}$$

$$N=\;\frac{n}{\mathrm{Prev}}$$

Whereas:

- Type 1 errors (*α*) = 0.05.
- Type 2 errors (*β*) = 0.2
- Hazard Ratio (HR) = 0.49 (*)
- Prevalence (Prev) = 0.57 (*)

(*): Sixt et al. (6) reported a HR of 0.49 with a TASC II A and B prevalence of 57% in 1,712 interventions. Using their formula, we aimed for 55 subjects or 31 events. However, anticipating a 20% sample loss due to various reasons (such as dropouts, non-compliance, etc.), we adjusted the sample to 69, ensuring an 80% completion rate. A total of 237 participants were initially recruited. Following the exclusion of 104 patients based on the study's criteria, 133 individuals remained for intervention and follow-up throughout the study period from October 2016 to September 2020, meeting the required number of participants. Notably, there was no participant loss during the follow-up period.

### Data collection tools

The study employed essential infrastructural resources at the Vascular Surgery Department, including handheld ABI devices and a computer with FPT software for measurements. Diagnostic tools like a GE E95 ultrasound (Chicago, IL) and Siemens SOMATOM Definition 128-slice CT Scanner (Erlangen, Germany) were used. The study utilized a digital Ziehm Vision R C-arm machine (Nuremberg, Germany), various medications, and emergency equipment at the Anesthesiology and Resuscitation Department.

### Procedure

#### Patient preparation

Patients received prophylactic antibiotics (Cefazoline, 1 g/bottle, two bottles intravenously) and were positioned with straight legs, exposing the abdomen, groin, thigh, and calf. Anesthesia options: local (for procedure <3 h) or endotracheal (for procedure >3 h or patients with contraindications of spinal anesthesia).

#### Intervention

The puncture site was sterilized and draped. Local anesthesia (2% Lidocaine) was given at the groin. Using ultrasound guidance, we inserted a short guidewire and sheath into the artery, followed by angiography to locate the lesion. Subsequently, we conducted balloon angioplasty following established guidelines. The procedure ended when residual lumen stenosis was under 30% without dissection; stents were placed otherwise. Patients received varied doses of heparin (50–100 mg/kg body weight) throughout the procedure. Afterward, we removed the guidewire and sheath, dressed the wound, and monitored and managed potential complications like vascular rupture, dissection, thrombosis, hematoma, and stent deformation.

#### Post-intervention assessment

In the peri-intervention assessment, we assessed various factors until discharge. This involves evaluating clinical symptoms like post-op pain, resting pain, pain at location (buttocks, thighs, calf, foot, toes), checking for non-healing ulcers, and gangrene. The Rutherford classification and ABI were also used. Subsequently, using ultrasound identifies normal states, stenosis, or occlusion.

The study involved short-term (1–12 months post-intervention) and medium-term (12 months until study end) follow-ups. Short-term focused on immediate responses and potential complications arising from the intervention, while medium-term assessed sustained effectiveness and late complications. We evaluated the patient's pre-intervention status using TASC II A and B classification. Technical assessment determined intervention success: <30% residual stenosis, no dissection (or dissection managed with immediate stent), and no stent kinks/fractures on post-op Digital subtraction angiography (DSA) for success. Failure means >30% residual stenosis, dissection, stent kinks/fractures seen, requiring further intervention or open surgery, or severe complications like limb loss or intervention-related death.

We clinically assessed the success by Rutherford category improvement: exhibiting one-category (intermittent claudication/resting pain) or two-category (tissue loss) improvement vs. pre-intervention. Failure is no or less than a two-category improvement, or potential complications include limb loss or intervention-related death. Complications monitored include arterial issues, hematoma at the entry site, stent deformation, organ failure, heart events, stroke, and limb loss.

### Statistical analysis

Data is collected using information collection forms, which is then cleaner, encoded, and processed. The data is entered using Excel software and analyzed using R 3.6.3 software. For truly missing values, we use the Imputation algorithm in R to fill in the blanks.

Quantitative variables are calculated for mean and standard deviation. The mean and standard deviation for age, ABI, balloon inflation pressure, intervention time, and hospital stay duration are reported. The frequency, and percentage are represented for qualitative variables.

### Ethics approval and informed consent to participate

The study obtained Ethical approval from the Ethical Committee of Ho Chi Minh City University of Medicine and Pharmacy (92/HDDD). Prior to data collection, the study subjects were provided with a clear explanation of the study's purpose and content. The study proceeded only after participants provided written consent, in which they agreed to participate voluntarily with the right to withdraw at any time and were guaranteed of being anonymous and kept confidential. Participants indicated their agreement by signing on the informed consent document.

## Results

### Characteristics of the patients prior to the intervention

Table 1 outlines baseline patient characteristics. The 133-participant sample had an average age of 70.2 ± 11.0 years, primarily aged 60–69 (33.8%). The cohort was predominantly male (85.0%), with an average BMI of 20.9 ± 2.67, and 77.4% had a normal BMI. Smoking affected 67.7%, and other prevalent comorbidities included hypertension (56.4%), carotid artery disease (37.6%), and diabetes (35.3%).

**Table 1:** Pre-intervention patient characteristics.

		Total (*N* = 133)	Percentage (%)
General characteristics			
Age (years) ± SD		70.2 ± 11.0	
<60		23	17.3
60–69		45	33.8
70–79		37	27.8
≥80		28	21.1
Gender	Male	113	85.0
	Female	20	15.0
BMI ± SD		20.9 ± 2.67	
<18.5		25	1,808
18.5–25		103	77.4
>25		5	3.8
Risk factors and comorbidities			
Smoking		90	67.7
Pack-year ± SD		20.6 ± 15.7	
Hypertension		75	56.4
Carotid artery disease		50	37.6
Diabetes		47	35.3
Prior cerebral stroke		17	12.8
COPD		16	12.0
Coronary artery disease		6	4.5
Atrial fibrillation		4	3.0
Chronic liver disease		3	2.3
Heart failure		3	2.3
Chronic kidney disease		2	1.5
Patient characteristics before intervention			
Intermittent claudication		60	45.1
Rest pain		73	54.9
Chronic ulceration		61	45.9
Necrotic gangrene		36	27.1
Infection		25	18.8
Pain location			
Soles		99	74.4
Legs		89	66.9
Toes		85	63.9
Thighs		48	36.1
Buttocks		23	17.3
Rutherford classification before intervention			
Asymptomatic		0	0
Class I		0	0
Class II		6	4.5
Class III		54	40.6
Class IV		11	8.3
Class V		50	37.6
Class VI		12	9.0
ABI before intervention		0.32 ± 0.20	
Mild (>0.75–0.9)		2	1.5
Moderate (0.4–0.75)		43	32.3
Severe (<0.4)		88	66.2
Duplex scan before intervention			
Common iliac artery			
Normal		55	41.4
Stenotic		54	40.6
Occluded		24	18.0
External iliac artery			
Normal		57	42.9
Stenotic		44	33.1
Occluded		32	24.1
Internal iliac artery			
Normal		130	97.7
Stenotic		1	0.8
Occluded		2	1.5
CTA before intervention			
Iliac artery calcification			
Normal		19	14.3
Mild		28	21.1
Moderate		39	29.3
Severe		47	35.3
Common iliac artery			
Normal		55	41.4
Stenotic		54	40.6
Occluded		24	18.0
External iliac artery			
Normal		57	42.9
Stenotic		44	33.1
Occluded		32	24.1
Internal iliac artery			
Normal		130	97.7
Stenotic		1	0.8
Occluded		2	1.5

SD, standard deviation; COPD, chronic obstructive pulmonary disease.

Pre-intervention, rest pain was experienced by 54.9% of patients, while 45.1% had intermittent claudication. Chronic ulceration, necrotic gangrene, and infection were reported in 45.9%, 27.1%, and 18.8% of patients, respectively. Pain mainly in soles (74.4%), legs (66.9%), toes (63.9%). The majority of patients were classified as Rutherford Class III (40.6%) or Class V (37.6%). The ABI was severely reduced (<0.4) in 66.2% of patients and moderately reduced (0.4–0.75) in 32.3%.

Duplex scan showed common iliac artery stenosis (40.6%) and occlusions (18.0%). Similar findings were seen in the external iliac artery with 33.1% stenotic and 24.1% occluded. The internal iliac artery was mostly normal (97.7%). As per Stoner et al. (7), calcification severity evaluated via Computed Tomography Angiography (CTA) is categorized into four groups: none, mild (<25% circumference), moderate (25%–50%), or severe (>50%). In our study, moderate to severe calcification on CTA was observed in 64.6% of the study population. Findings in common and external iliac arteries aligned with duplex scan results. The internal iliac artery mainly appeared normal, as observed in 97.7% of cases.

### TASC II classification of the study population

In the TASC II classification of 133 patients with balloon angioplasty or stenting, 73.9% of balloon angioplasty recipients fell under TASC II Class A, while 63.2% of stenting patients were Class B. Overall, the cohort was evenly split between Class A (49.6%) and Class B (50.4%), showing a balanced distribution (Table 2).

**Table 2:** TASC II classification of the study population.

Characteristics		Balloon *n* = 46 (%)	Stenting *n* = 87 (%)	Total *n* = 133 (%)
TASC II classification	A	34 (73.9)	32 (36.8)	66 (49.6)
	B	12 (26.1)	55 (63.2)	67 (50.4)

### Procedure characteristics

Table 3 provides an overview of the intervention characteristics. Out of the 133 subjects, the majority of interventions (89.5%) were conducted under local anesthesia, with an average intervention duration of 150 ± 51.5 min, and an average contrast volume of 50.9 ± 10.2 ml. Patients typically remained in the hospital for an average of 4.7 ± 2.7 days post-intervention.

**Table 3:** Characteristics of the intervention for the study subjects.

Characteristics	Total (*N* = 133)	Percentage (%)
General anesthesia	14	10.5
Local anesthesia	119	89.5
Time of intervention (minutes) ± SD	150 ± 51.5	
Amount of contrast (ml)	50.9 ± 10.2	
Hospitalization period (days)	4.7 ± 2.7	
Characteristics of intervention		
Access site		
Contralateral femoral artery	61	45.9
Ipsilateral femoral artery	56	42.1
Brachial artery	13	9.8
Popliteal artery	3	2.2
Intervened artery		
Common iliac artery	51	38.3
External iliac artery	59	44.4
Both	23	17.3
Associated interventional artery		
Femoral artery	84	63.2
Interventional method		
Balloon angioplasty	46	34.6
Stenting	87	65.4
Balloon types (*n* = 46)		
Non-drug coated	46	100
Balloon diameter (mm) (*n* = 46) ± SD	6.5 ± 0.7	
Balloon length (mm) (*n* = 46)		
60 mm	0	0.0
80 mm	7	15.2
100 mm	19	41.3
120 mm	14	30.4
150 mm	6	13.1
Types of stents (*n* = 87)		
Self-expanding	81	93.1
Balloon-expanding	6	6.9
Stent diameter (mm) ± SD	7.3 ± 0.6	
Stent length (*n* = 87)		
60 mm	1	1.2
80 mm	24	27.6
100 mm	35	40.2
120 mm	19	21.8
150 mm	8	9.2
Number of stents		
One	86	98.9
Two	1	1.1

For intervention specifics, access was mainly through contralateral (45.9%) or ipsilateral femoral artery (42.1%). The intervened arteries were predominantly the common iliac (38.3%) and external iliac artery (44.4%), with both being intervened in 17.3% of cases. Additionally, the femoral artery was utilized as an additional intervention in 63.2% of cases.

In terms of the intervention method, 34.6% of cases underwent balloon angioplasty, while 65.4% received stents. Balloon angioplasty used non-drug coated balloons, averaging 6.5 ± 0.7 mm diameter, most commonly 100 mm long (41.3%). Stenting involved 93.1% self-expanding stents, averaging 7.3 ± 0.6 mm diameter, with a preferred length of 100 mm (40.2%). Nearly all (98.9%) received a single stent.

### Peri-intervention outcomes

Table 4 shows immediate post-intervention outcomes. Technical success reached 97.7%, while clinical success was 62.4%. Three instances of technical failures were observed: two cases necessitated above-the-knee amputation at 3 days and 25 days post-procedure, respectively, and one case experienced thrombosis resulting in distal embolisation of the common iliac artery, necessitating open surgery for blood clot removal. Notably, all three of these cases occurred in patients who underwent stent placement.

**Table 4:** Peri-intervention outcomes of the study subjects.

Characteristics	Total (*N* = 133)	Percentage (%)
Immediate post-intervention treatment results		
Technique		
Successful	130	97.7
Failed	3	2.3
Clinical		
Successful	83	62.4
Failed	50	37.6
Intervention complications		
Major limb amputation	2	1.5
Thrombosis	1	0.8
Access site hematoma	1	0.8
Acute kidney failure	1	0.8
Myocardial infarction	1	0.8
Additional surgery	1	0.8
Stent deformation	0	0
Vessel rupture	0	0
Cerebral stroke	0	0
Additional intervention	0	0
Death	0	0

Other complication occured in ≤1% of cases each—access site hematoma (1 case), acute kidney failure (1 case), myocardial infarction (1 case).Notably, no instances of stent deformation, vessel rupture, stroke, further intervention, or death were reported.

### Short-term and medium-term outcomes of the study population

Table 5 presents the outcomes of post-intervention follow-up at both short-term and mid-term intervals. The study encompassed short-term (1–12 months post-intervention) and medium-term (12 months until the end of the study) follow-ups. The average follow-up duration was 37.2 ± 1.3 months, ranging from 1 to 45 months.

**Table 5:** Short-term and mid-term outcomes of the study subjects.

Characteristics	Short-term outcomes		Mid-term outcomes	
	Total (*N* = 133)	Percentage (%)	Total (*N* = 115)	Percentage (%)
Rutherford classification				
Asymptomatic	17	12.8	16	13.9
Class I	56	42.1	51	44.3
Class II	41	30.8	36	31.3
Class III	15	11.2	10	8.7
Class IV	1	0.8	1	0.9
Class V	0	0	0	0
Class VI	1	0.8	0	0
Non-assessed	2	1.5	1	0.9
ABI** **± SD	0.76 ± 0.2		0.76 ± 0.2	
Mild (>0.75–0.9)	87	65.4	75	65.2
Moderate (0.4–0.75)	39	29.3	33	28.7
Severe (<0.4)	5	3.8	6	5.2
Non-assessed	2	1.5	1	0.9
Patency				
Yes	120	90.2	99	86.1
No	13	9.8	16	13.9
Follow-up complications				
Major limb amputation	0	0	1	0.9
Cerebral stroke	3	2.3	3	2.6
Death				
Arterial occlusion-related	3	2.3	2	1.7
Non-related arterial occlusion	15	11.3	7	6.1
Re-stenosis	6	4.5	16	13.9

**Figure 2:**
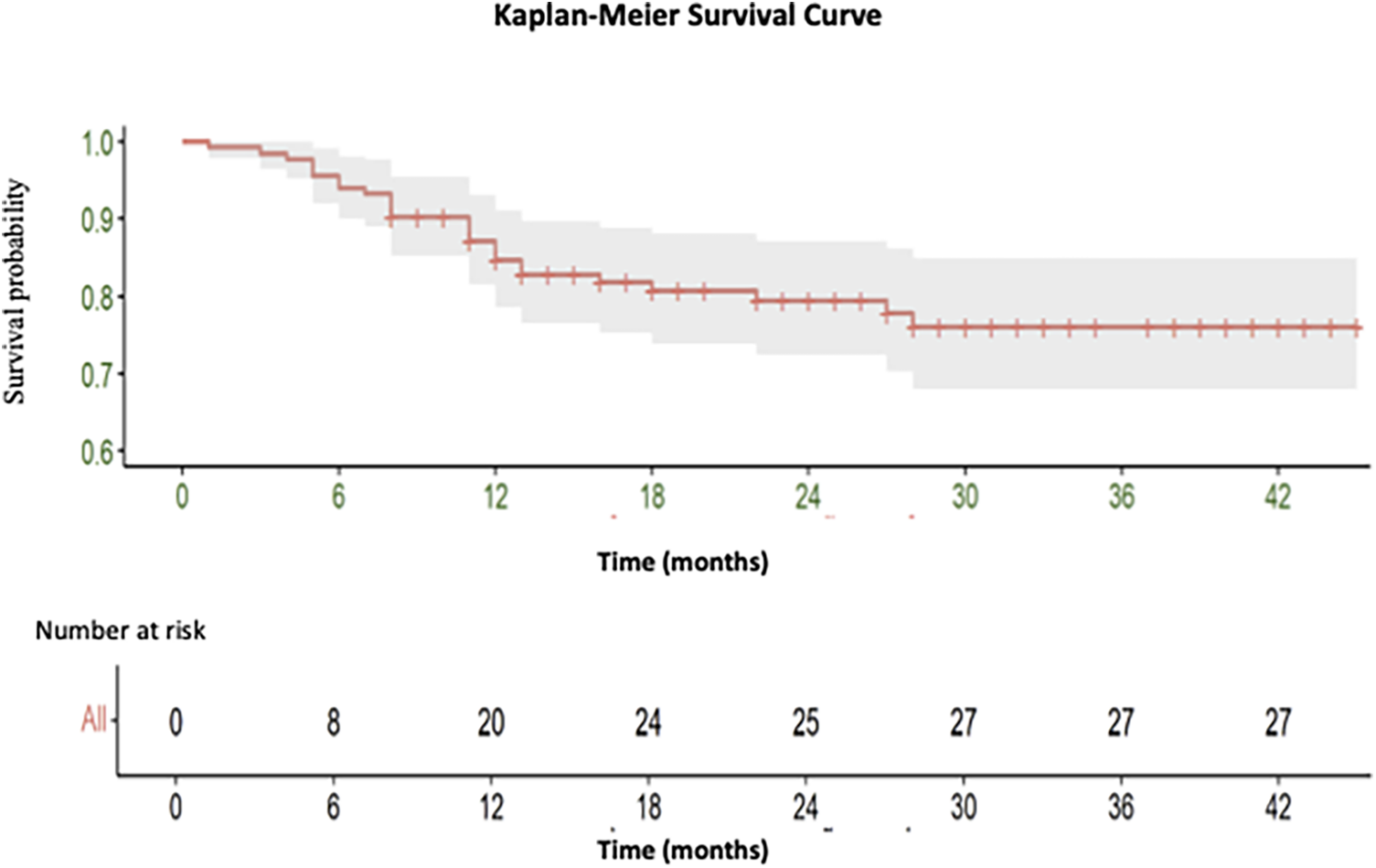
Kaplan-Meier curve illustrating the overall survival of endovascular intervention of iliac artery lesions classified as TASC II A and B.

A total of 27 deaths were recorded, with 18 occurring during the short-term follow-up and 9 during the medium-term follow-up period [See (Figure 2)].

#### Short-term outcomes (*n* = 133)

In the short-term period, 12.8% of patients were asymptomatic, andmost fell within the first three Rutherford categories (I, II, III) at 42.1%, 30.8%, and 11.2%, respectively. Severe symptoms were rare, including 0.8% at stage IV and 0.8% at stage VI. The average post-intervention ABI was 0.76 ± 0.2, indicating a general improvement. The majority of patients (65.4%) had mild severity as per ABI (≥0.75), while 29.3% and 3.8% had moderate (0.4–0.75) and severe (<0.4) ABI scores, respectively. Vascular circulation post-intervention was successful in 90.2% of patients, demonstrating a generally positive outcome following the intervention. Notably, there were three instances of death attributed to arterial occlusion, accounting for 2.3% of the total cases, and 15 deaths due to non-related arterial occlusion, constituting 11.3% of the cases.

#### Mid-term outcomes (*n* = 115)

In the mid-term post-intervention phase, 13.9% of patients were asymptomatic. The majority of patients were classified under the first three Rutherford stages (I, II, III), with proportions of 44.3%, 31.3%, and 8.7% respectively. Only one patient presented more severe symptoms, with 0.9% at stage IV, and none at stages V and VI. Post-intervention ABI averaged 0.76 ± 0.2, showing sustained improvement Patients with a mild score (≥0.75) comprised 65.2% of the sample, while those with moderate (0.4–0.75) and severe (<0.4) ABI scores accounted for 28.7% and 5.2%, respectively. The status of vascular circulation remained largely positive, with successful circulation observed in 86.1% of patients, while unsuccessful in 13.9%. This indicates a generally positive trend in the patient's recovery at the mid-term follow-up. Notably, two deaths (1.7%) were attributed to arterial occlusion, and seven cases (6.1%) were due to non-related arterial occlusion.

## Discussion

This study was conducted between October 2016 and September 2020 and a total number of 133 cases were involved. The average age was 70.2 ± 11.0 years. Comparable to prior research, Zang and Li et al. found a mean age of 64.04 ± 10.7 in their endovascular treatment study (8). Likewise, two other studies in Vietnam reported a similar age distribution (9, 10).

The age and gender distribution in our study align with the broader demographic trend observed in patients with chronic iliac artery occlusion, predominantly affecting older males. Comparable studies have also demonstrated similar male majorities, ranging from 73% to 88% (8, 10, 11). This dominance could be attributed to various risk factors, potentially including lifestyle choices and genetic predispositions that may disproportionately affect males. Despite male predominance, women's susceptibility to PAD rises post-menopause, underscoring the importance of tailored preventive strategies (12, 13).

In our cohort, smoking emerged as the predominant risk factor, consistent with previous studies highlighting its significance (14, 15). Its strong link to PAD is well-documented in studies like Weiming Wang et al. (16). Thus, our findings further emphasize the role of smoking as a critical risk factor in the development and progression of PAD.

Majority of the patients in the study had a high pre-intervention Rutherford classification with Grade III being the highest. Pulli et al. made a similar observation—having 91.05% of participants in Rutherford grade III (15). Our average intervention time for TASC II A/B, 150 ± 51.5 min, aligns with Shigeo Ichihashi et al.'s study (17).

In our study the access was achieved primarily through the contralateral femoral artery (45.9%), ipsilateral femoral artery (42.1%), brachial artery (9.8%), and the popliteal artery (2.2%). In Pulli et al. the access was through the ipsilateral artery in most cases (70%), 15% in the contralateral artery, 10.5% opened ipsilateral femoral artery and brachial artery was 4.5% (15). Lakhter et al. postulated that the position of access can be via ipsilateral retrograde common femoral artery (CFA), contralateral antegrade CFA or even via brachial artery route depending on the structure of the lesion, as well as the location of the distal and proximal stumps (18).

Balloon dilation was used in 34.6% of cases, while stents were used in 65.4% of cases. The overall technical success rate of the procedure was 97.7%. Balloon dilatation had a technical success rate of 100%. The high success rates observed in our study for both balloon dilation and stenting emphasize their efficacy in treating chronic iliac artery occlusions. The marginally lower success rate for stenting, however, may be reflective of the inherent complexities associated with this procedure, including technical difficulties and patients' health status. This study illustrates that angioplasty without stenting yielded favorable outcomes, diverging from the recommendation of primary stent implantation for aorto-iliac lesions as per the guidelines of the European Society for Vascular Surgery (ESVS) (19). Our study found that endovascular stenting demonstrated a high success rate of 97.7%. This aligns well with previous studies conducted in this field (20–22).

These findings, collectively, affirm the robust efficacy of endovascular stenting as a treatment strategy for peripheral arterial occlusions.

A recent systematic review and meta-analysis found no evidence of a difference between the two groups in technical success rates, whether in studies involving stenotic lesions or those involving iliac artery occlusions. Notably, only one trial reported a higher rate of major complications, particularly distal embolization, with balloon angioplasty of iliac artery occlusions (23). In our study, technical success reached 97.7%. Three instances of technical failures were observed: two cases required above-the-knee amputation, while one case experienced distal embolization of the common iliac artery. Remarkably, all three of these cases occurred in patients who underwent stent placement. It's essential to note that our study primarily aimed to evaluate the short and medium-term outcomes of overall endovascular intervention, encompassing both balloon angioplasty and stent placement, for treating iliac artery lesions categorized as TASC II A or B. Future studies may benefit from reporting procedural details and resulting patency rates separately for each method. This approach could offer a more comprehensive understanding of which technique yields superior outcomes for specific lesion presentations.

In terms of interventional complications, our study identified a total of 6 cases, constituting 4.5% of the cohort. These complications included two instances of major limb amputation, one occurrence of thrombosis necessitating additional surgery, one case of access site hematoma, one case of acute renal failure, and one instance of myocardial infarction. These findings echo the findings of Shigeo Ichihashi et al., who reported a procedural complication rate of 4.8%, with distal embolism and pseudoaneurysm being the most prevalent complications (17). The complication rate found in our study is also congruent with rates reported in other studies, as evidenced in a systematic review by Vincent Jongkind et al. (24) This emphasizes the importance of being cognizant of these potential complications in the management of patients undergoing endovascular procedures for iliac artery occlusions.

Following the intervention, we observed a marked improvement in the patients' symptoms. Notably, the patients reported alleviation in pain, warmer extremities, and enhanced mobility. Moreover, indications of non-healing ulcers, necrosis, and infections showed considerable improvement. This improvement is reflected in the significant changes in Rutherford grades V and VI both in the short term and medium term. Prior to the intervention, these grades accounted for 37.6% and 9%, respectively. Post-intervention, these figures dropped to 0% and 0.8% in the short term and 0% for both grades in the medium term.

Galaria et al. reported comparable improvements, with an average clinical symptom score decreasing from 3.3 ± 0.9 before intervention to 2.1 ± 0.7 post-intervention (22). Furthermore, they noted that 84% of patients experienced an improvement in their clinical symptoms. This percentage aligns with the findings of Sullivan et al., who also reported an improvement in clinical symptoms among 84% of patients following intervention (25). These consistent findings across multiple studies underscore the effectiveness of endovascular interventions in improving symptoms related to peripheral artery disease.

Our study noted an appreciable increase in the ABI, from a preoperative measure of 0.3 ± 0.2–0.76 ± 0.2 in both the short and medium-term follow-up periods. This improvement in ABI suggests enhanced hemodynamics following the intervention. Furthermore, we recorded 13 instances of non-vascular circulation in the short-term period, and 16 in the medium-term period. This translates to vascular circulation rates of 90.2% and 86.1% in the short and medium term respectively. These rates are corroborated by comparable studies such as those by Shigeo Ichihashi et al., who reported short-term and mid-term vascular rates of 97% and 84% respectively (17), and Min Yang et al., who noted similar rates of 98.6% and 90.1% for the short and mid-term periods (26).

In our study, the mortality rate attributable to arterial occlusion was observed to be 2.3% in the short term and slightly less at 1.7% over the mid-term. Deaths not related to occlusion accounted for 11.3% in the short term and decreased to 6.1% in the mid-term. Notably, these mortality rates were lower compared to those reported in related studies. Specifically, studies by Kasemi et al. and Soares et al., which both examined the outcomes of iliac artery interventions, reported higher mortality rates of 4.5% and 4.3% respectively (27, 28).

Our study observed restenosis rates of 4.5% in the short term, which rose to 13.9% over the longer term. When compared with similar studies, our results align reasonably. For instance, the multicenter study by Palmaz reported a rate of 8% at 9 months, while Walters' study observed a restenosis rate of 12% at the 6-month mark (29, 30). Further down the timeline, the study by Davies revealed a 15% restenosis rate at the 5-year mark, and Schurmann et al. reported a restenosis incidence of 21% after 6 years (31, 32).

The authors wish to underscore that while our study predominantly focused on evaluating the outcomes of interventions directed at chronic iliac artery occlusions, it is imperative to consider the broader vascular context when devising optimal therapeutic strategies for patients presenting with multifocal lesions extending beyond the iliac arteries. Notably, our study cohort exhibited a notable prevalence of rest pain (55%) and necrotic gangrene (27%), indicative of multifocal disease extending beyond the confines of the iliac arteries. Following stenting of the common iliac, external iliac, or both arteries (supplemented by femoral artery intervention as necessary), only one patient (0.9% of the total cases) necessitated amputation, while none reported rest pain in the mid-term outcomes, suggesting a favorable outcome following iliac artery intervention in the context of multifocal disease. Nevertheless, comprehensive, large-scale studies are warranted to validate and confirm such findings.

## Limitations

While the study provides crucial insights, it is important to acknowledge its limitations. The single-center design could limit the generalizability of the findings to other populations. Furthermore, the sample size, although considerable, may not be large enough to detect differences in less common outcomes or complications.

## Conclusion

The study highlights the favorable short-term and medium-term outcomes following the intervention, underscoring the efficacy of balloon dilation and stent placement for treating TASAC II A/B iliac artery stenoses or occlusions. It encourages further exploration into extended-term effects, advocating larger, diverse longitudinal studies to enhance treatment strategies and patient outcomes while understanding potential complications.

## Data Availability

The raw data supporting the conclusions of this article will be made available by the authors, without undue reservation.
